# P-89. Impact of Treatment of Positive Fungal Cultures in Diabetic Foot Infections

**DOI:** 10.1093/ofid/ofaf695.318

**Published:** 2026-01-11

**Authors:** Laila M Castellino, Alexander M Tatara, Michael Fanning, Freeman Brunaugh, Elizabeth Haddad, John Hanna, Arden Harada, Francesca Lee, Andrew E Clark, Peter Crisologo

**Affiliations:** University of Texas Southwestern Medical Center, Dallas, TX; University of Texas Southwestern Medical Center, Dallas, TX; University Of Texas Southwestern Medical Center Dallas, TX, Dallas, Texas; University Of Texas Southwestern Medical Center Dallas, TX, Dallas, Texas; Parkland Health, Dallas, Texas; University Of Texas Southwestern Medical Center Dallas, TX, Dallas, Texas; University of Texas Southwestern, Dallas, TX; University of Texas Southwestern Medical Center, Dallas, TX; University Of Texas Southwestern Medical Center Dallas, TX, Dallas, Texas

## Abstract

**Background:**

The significance of Candida species and other fungi isolated in diabetic foot infections (DFI) is unclear. Do these isolates require treatment? Antifungal treatment carries additional cost and risk of adverse events. In this study, we compared outcomes among patients with DFI that were treated vs. not treated for fungi isolated from surgical specimens.

**Figure:**
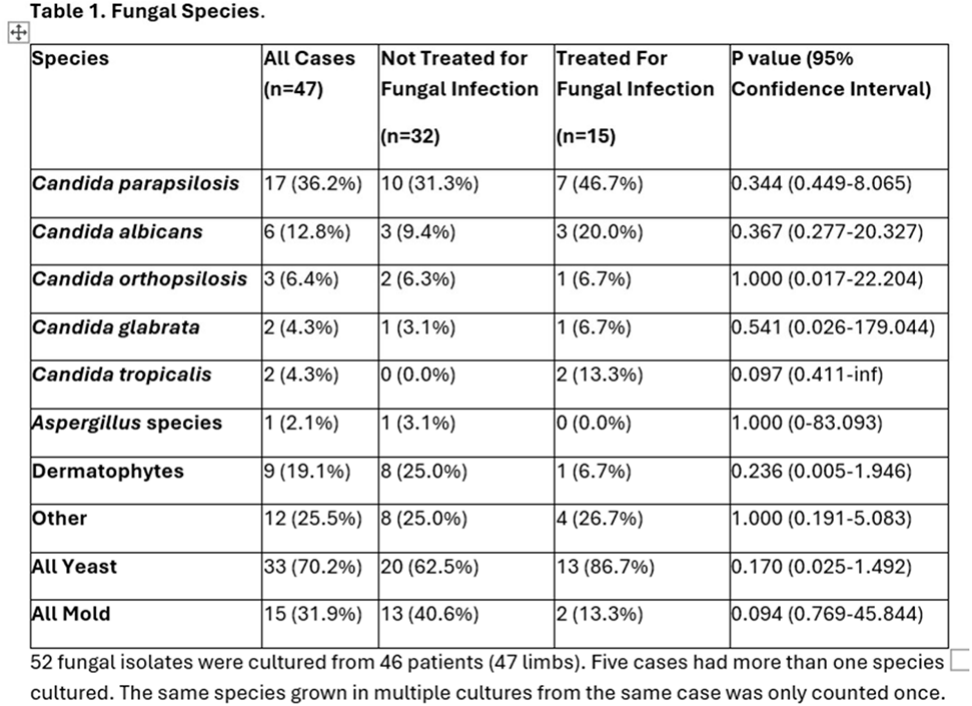

**Figure:**
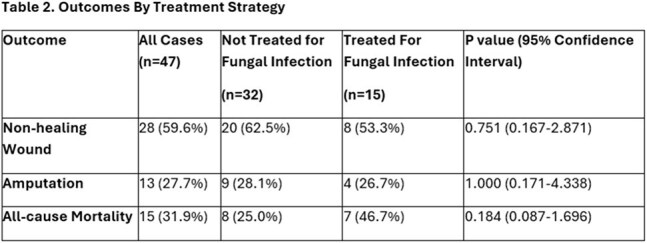

**Methods:**

We conducted a retrospective review of adults undergoing surgery for DFI with surgical cultures positive for fungi from Oct 1, 2019 to Sept 30, 2022, at a teaching hospital in Dallas, Texas. Patient outcomes were assessed up until last recorded visit or 12 months post-surgery. Descriptive statistics were used to describe the study cohort using medians and interquartile range (IQR) for continuous variables and percentages for categorical variables. To compare outcomes among treated versus untreated patients, Fisher’s Exact Test was used with α=0.05 (R v4.4.1).

**Results:**

Forty-seven limbs from 46 patients had positive fungal cultures. Median age was 59 years, 68 % male, 62% white, 30% Hispanic, median HgA1c was 7.8%, with 52% having excisional debridements, 19% toe amputation, 13 % ray amputation, 15% transmetatarsal amputation and 2% above or below-knee amputation. Sixty percent of all cases had non-healing wounds. Yeast grew in 70% of specimens, with *C. parapsilosis* being most common (36%), followed by *C. albicans* (13%). Mold grew in 32% of specimens including dermatophytes in 19% of specimens. Most patients (68%) were not treated for fungal infection. When comparing patients treated vs. untreated for positive fungal cultures, there were no statistically significant differences in wound healing (53% vs 62%), subsequent amputation (27% vs 28%,) or all-cause mortality (47% vs 25%).

**Conclusion:**

Our findings did not demonstrate significant differences in outcomes among patients that were or were not treated for positive fungal cultures in patients undergoing surgery for DFI. Compared to prior DFI studies, patient mortality rates were similar although rates of non-healing wounds were higher. While outcomes in DFI are dependent on multiple factors, further research is needed to determine the need/added value of treatment for fungal organisms isolated from surgical specimens in DFI.

**Disclosures:**

All Authors: No reported disclosures

